# MYCN contributes to the malignant characteristics of erythroleukemia through EZH2-mediated epigenetic repression of p21

**DOI:** 10.1038/cddis.2017.526

**Published:** 2017-10-12

**Authors:** Li Liu, Feng Xu, Chun-Kang Chang, Qi He, Ling-Yun Wu, Zheng Zhang, Xiao Li

**Affiliations:** 1 Department of Hematology, Shanghai Jiao Tong University Affiliated Sixth People's Hospital Shanghai 200233, China

## Abstract

MYC proto-oncogene family including c-myc and n-myc (MYCN) are critical for normal cell development and tumorigenesis. Overexpression of c-myc causes acute erythroleukemia *in vivo*. However, the role of MYCN in acute erythroleukemia remains poorly understood. In this study, we found that the patients with erythroleukemia showed higher expression of MYCN than normal controls. *In vitro* experiments, knockdown of MYCN resulted in decreased cell proliferation, elevated autonomously cell apoptosis and increased P21-mediated cell senescence. On the contrary, overexpression of MYCN obviously promoted cell proliferation, and induced erythroid differentiation block and apoptosis resistance to cytotoxic agent. Further gene microarray and functional analysis revealed that EZH2 is a target of MYCN. Knockdown of MYCN inhibited the expression of EZH2, and then activated p21 expression through removal of H3K27me3 at the p21 promoter. Overexpression of ezh2 could antagonize the p21 activation caused by MYCN knockdown. In addition, Aurora inhibitor MLN8237 inhibited the proliferation of erythroleukemia cells through repression of MYCN/EZH2 axis, whereas it minimally affected the normal hematopoietic cells. In conclusion, MYCN contributes to the malignant characteristics of erythroleukemia through EZH2-meidated epigenetic repression of p21. MYCN may serve as a therapy target for the patients with acute erythroleukemia.

MYC proto-oncogene family, comprising c-myc (MYC), n-myc (MYCN) and l-myc (MYCL), are critical for normal cell development and proliferation.^1^ Abnormal expression of MYC family promotes the tumorigenesis in multiple human cancers.^2^ MYC is one of the most common oncogenes in human cancers, and frequently associated to lymphoma and lymphoblastic leukemia.^2, 3^ Increasing evidence has showed that MYC also has a driving role in myeloid malignancies.^4, 5, 6^ MYC in the context either of Arf/Ink4a loss or Bcl-2 overexpression induced a mixture of acute myeloid and acute lymphoid leukemia.^4^ Collaboration of MYC with GATA-1 could induce an erythroleukemia in mice.^5^ MYC cooperates with BCR-ABL to drive chronic myeloid leukemia progression to acute myeloid leukemia (AML).^6^ However, the role of MYCN in AML remains poorly understood.

MYCN gene located at chromosome 2p24.3 was first identified in neuroblastoma cell lines as amplified DNA with homology to viral MYC.^7^ Similar to the MYC, MYCN has a conserved structure including a transcriptional activation domain in the N terminus and a C-terminus basic helix-loop-helix leucine zipper domain, which binds specific DNA sequence and regulates gene transcription.^8^ The role of MYCN in tumorigenesis is mainly investigated in neuroblastoma.^9^ MYCN gene is amplified and associated with poor prognosis in neuroblastoma.^9^ In addition, MYCN amplification or overexpression has been shown in several other cancers, including small cell lung cancer, prostate cancer and Wilms tumor.^10, 11, 12^ However, few studies were performed to investigate the role of MYCN in hematopoietic malignancies. Transgenic MYCN expression induced lymphoma in mouse model.^13^ Overexpression of MYCN was observed in some patients with acute myeloid leukemia.^14^ Leukemia mouse model also showed elevated MYCN expression.^15^ All these studies suggest that MYCN may be vitally critical for leukomogenesis.

Acute erythroleukemia (AML-M6) is an uncommon subtype of AML with a worse prognosis. Considering the pivotal role of MYC in erythroleukemia development, we explored the biological function of MYCN in erythroleukemia cell lines HEL and K562. The mechanism of MYCN in maintenance of malignant characteristic of leukemia cells was investigated by cell functional assays, gene microarray, and chromatin immunoprecipitation.

## Results

### MYCN is overexpressed in the patients with erythroleukemia

**Figure 1:**
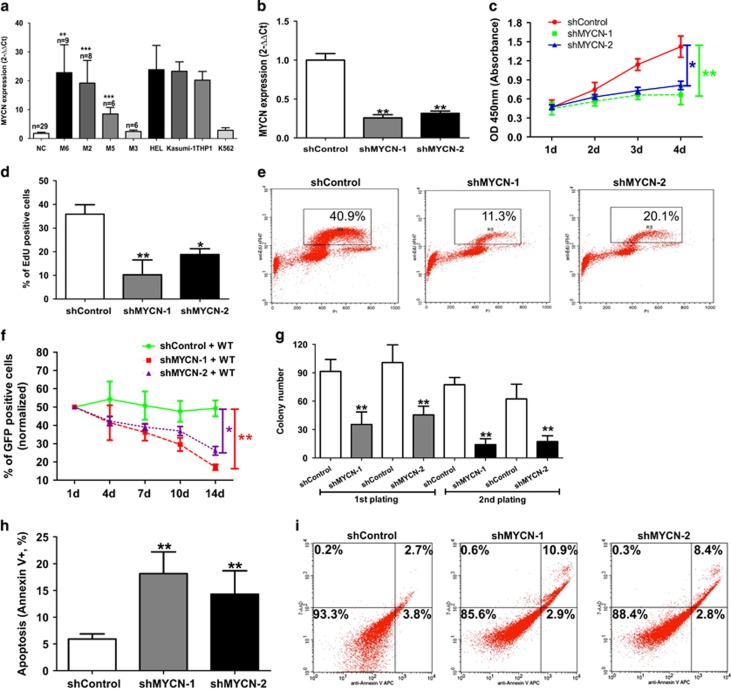
MYCN is indispensable for the maintenance of malignant phenotypes of erythroleukemia cell line. (**a**) Higher MYCN expression was found in the AML-M6, M2 and M5 compared with the normal controls (all *P*<0.01). (**b**) The expression of MYCN mRNA (left, determined by qRT-PCR) (*P*=0.008) and protein (right, determined by FCM) was significantly reduced after transfection with shMYCN. (**c**) WST-1 assay indicated that HEL cells with MYCN knockdown showed reduced cell growth rate compared with the controls cells (*P*=0.006). (**d**) Knockdown of MYCN resulted in a significantly reduced cell percentage with EdU-positive staining (*P*=0.003) (left). Representative FCM chart was shown (right). (**e**) Competing co-culture showed that the percentage of cells with MYCN depletion (indicated by GFP) reduced gradually along with prolonged co-culture time (*P*=0.002). (**f**) Reduced plating and serial replating capacity was observed in the MYCN-knockdown cells compared with the control cells (all *P*<0.01). (**g**) Knockdown of MYCN resulted in an increased autonomous cell apoptosis in HEL cells (left) (*P*=0.008). Representative FCM chart was shown (right). Every assay was repeated three times. The representative graphic is shown. The corresponding statistical analysis relative to the control group is annotated with an asterisk. *P<0.05; **P<0.01; ***P<0.001 (two-tailed, student *t*-test). Error bars throughout represent the S.E.M.

MYCN expression was significantly higher in the erythroleukemia patients compared with the normal controls (*P*=0.003) (Figure 1a). MYCN expression was also significantly higher in the patients with M2 or M5 (*P*<0.001; *P*<0.001) compared with the normal controls. Similar to MYCN expression, MYC expression was also higher in the patients with erythroleukemia, M2 or M5 (all *P*<0.05) compared with the normal controls (Supplementary Figure S1a). MYCL expression was significantly lower in the patients with erythroleukemia compared with the normal controls (*P*=0.011) (Supplementary Figure S1b).

### MYCN is indispensable for the maintenance of malignant phenotypes of erythroleukemia cell line

In order to investigate the role of MYCN in malignant characteristics of erythroleukemia, we used erythroleukemia cell lines HEL and K562 to perform a series of functional experiments. High and moderate expression of MYCN is observed in the HEL and K562 cells compared with the normal controls, respectively (Figure 1a). We established HEL cells with stable knockdown of MYCN by using a lentivirus-mediated transfection system. QRT-PCR showed over 70% decrease in the expression of MYCN (shMYCN-1, 74% shMYCN-2, 71%) (Figure 1b). To evaluate the proliferation dependence of HEL cells on MYCN, we performed WST-1 proliferation assay, EdU uptake assay and growth fitness assay. WST-1 assay indicated that HEL cells with MYCN knockdown showed reduced cell growth rate compared with the controls cells (Figure 1c). EdU uptake assay showed that HEL cells with MYCN knockdown had obviously decreased cell percentage with EdU-positive compared with the control cells after treatment with 20 *μ*M EdU for 30 min (Figure 1d). Representative FCM charts were shown (Figure 1e). We also measured the percentage of GFP positive cells (indicating MYCN depletion) to observe the effect of MYCN on cell proliferation over time through co-culture of transfected HEL cells (shMYCN or shControl) with wild-type HEL cells. The percentage of cells with GFP positive reduced gradually, indicating that the cells with MYCN depletion showed worse competing survival (Figure 1f).Together, these assays revealed that knockdown of MYCN led to impaired cell proliferation ability in the HEL cells. Furthermore, methylcellulose assay was used to evaluate the effect of MYCN on colony formation. Reduced plating and serial replating capacity was observed in the MYCN-knockdown cells compared with the control cells (Figure 1g). We also observed that knockdown of MYCN resulted in an increased autonomous cell apoptosis in HEL cells (Figure 1h). Representative FCM charts were shown (Figure 1i). In addition, we also investigated the effect of MYCN on cell biological behaviors in non-erythroleukemia cell line Kasumi-1. MYCN-knockdown Kasumi-1 cells were constructed using lentivirus-mediated transfection (Supplementary Figure S2a). Suppression of MYCN inhibited cell proliferation, reduced the colony formation, induced cell cycle arrested into G0/G1 phase and led to increased cell apoptosis (Supplementary Figure S2b-e). Taken together, all these data suggest that MYCN is indispensable for the maintenance of malignant phenotypes of erythroleukemia.

### Overexpression of MYCN promotes cell proliferation, enhances the resistance to etoposide-induced cell apoptosis and induces erythroid differentiation block

**Figure 2:**
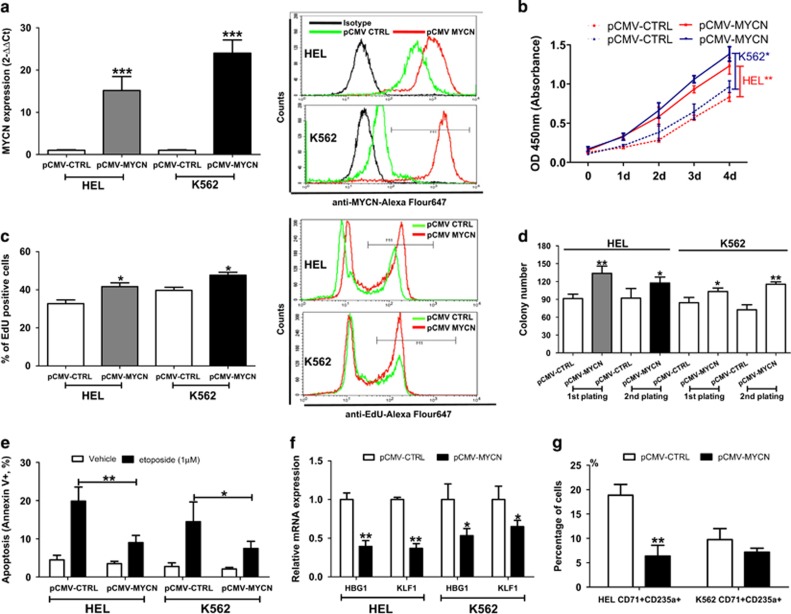
Overexpression of MYCN promotes cell proliferation, enhances the resistance to etoposide-induced cell apoptosis and induces erythroid differentiation block. (**a**) The expression of MYCN mRNA (left, determined by qRT-PCR) and protein (right, determined by FCM) was significantly overexpressed in HEL and K562 after transfection with pCMV-MYCN lentivirus. (**b**) Overexpression of MYCN resulted in a significantly elevated cell growth in HEL (*P*=0.009) and K562 (*P*=0.027) cells. (**c**) Overexpression of MYCN resulted in a significantly elevated cell percentage with EdU-positive staining in HEL (*P*=0.024) and K562 (*P*=0.033) cells (left). Representative FCM chart was shown (right). (**d**) Methylcellulose assay indicated that the HEL and K562 cells with MYCN overexpression showed enhanced plating and serial replating capacity compared with control cells (all *P* < 0.05). (**e**) MYCN overexpression resulted in reduced cell apoptosis sensitivity to etoposide in HEL (*P*=0.008) and K562 (*P*=0.029) cells. (**f**) Erythroid differentiation in myeloid cell lines with MYCN overexpression treated with 50 *μ*M hemin, as measured by HBG1 and KLF1 expressions relative to the control cells. The expression of HBG1 and KLF1 was obviously reduced in HEL and K562 cells with MYCN overexpression compared with the control cells (all *P*<0.05). (**g**) Percentage of CD71+CD235a+ populations in HEL with MYCN overexpression was obviously reduced compared with the control cells (*P*=0.004). Percentage of CD71+CD235a+ K562 cells with MYCN overexpression was also reduced although no difference was observed

Meanwhile, we constructed HEL and K562 cells with stable overexpression of MYCN by using a lentivirus-mediated transfection system. QRT-PCR and FCM analysis showed significantly increased expression of MYCN in the transfected HEL and K562 cells (Figure 2a). Upregulation of MYCN expression also conferred HEL and K562 cells to significantly increased growth capacity (Figure 2b). Further EdU uptake assay confirmed the promotion of MYCN overexpression on the proliferation of HEL and K562 cells (Figure 2c). Methylcellulose assay indicated that the HEL and K562 cells with MYCN overexpression showed enhanced plating and serial replating capacity compared with control cells (Figure 2d). It is well known that MYC-amplication tumor cells could overcome apoptosis and obtain a proliferative advantage.^16^ To understand the role of MYCN in cytotoxic agents-induced cell apoptosis, we investigated the effect of MYCN on apoptosis sensitivity of HEL and K562 cells to etoposide. As shown in Figure 2e, we observed that MYCN overexpression resulted in reduced cell apoptosis sensitivity to etoposide in HEL and K562 cells. MYCN overexpression was also constructed in Kasumi-1 (Supplementary Figure S2f). MYCN overexpression promoted cell proliferation, increased the colony formation and reduced cell apoptosis sensitivity to etoposide (Supplementary Figure S2g-2k). HEL and K562 cells have a capacity with erythroid differentiation. To investigate the effect of MYCN on erythroid differentiation, we co-cultured both of transfected cell lines with 50 *μ*M hemin or 3U/ml EPO for 72h to induce erythroid differentiation. We assessed the expression of the erythroid differentiation markers HBG1 and KLF1 using qRT-PCR. The expression of HBG1 and KLF1 was obviously reduced in HEL and K562 cells with MYCN overexpression compared with the control cells (Figure 2f and Supplementary Figure S3a). In addition, MYCN overexpression led to a significant reduction in the percentage of hemin-induced or EPO-induced CD71+CD235a+ erythroid populations in HEL and K562 cells (Figure 2g and Supplementary Figure S3b). These results suggests that overexpression of MYCN promotes malignant transformation.

### Depletion of MYCN induces p21-mediated cell senescence in a p53-independent manner

**Figure 3:**
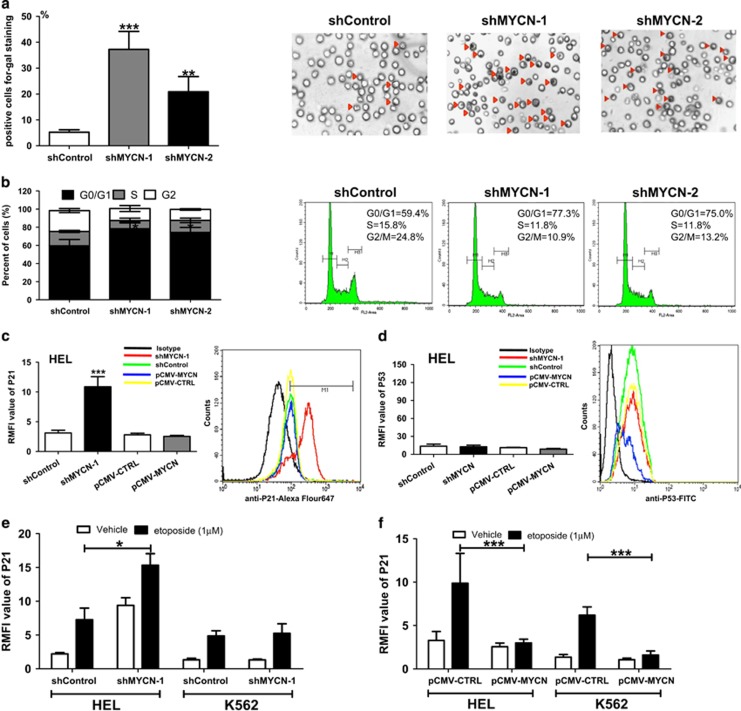
Depletion of MYCN induces p21-mediated cell senescence in a p53-independent manner. (**a**) Suppression of MYCN resulted in an increase in the percentage of senescent cells (*P*<0.001) (left). Representative cell senescence chart (shControl vs. shMYCN) was shown (right). Red triangle indicates the senescent cell. (**b**) Cell cycle arrested in G0/G1 phase was observed in the MYCN-knockdown cells (*P*=0.017). Representative cell cycle chart (shControl vs. shMYCN) was shown (right). (**c**) Knockdown of MYCN resulted in an increase in P21 expression in HEL cells (*P*<0.001). Representative FCM chart on P21 expression was shown (right). (**d**) Knockdown of MYCN did not result in an increase in P53 expression in HEL cells. Representative FCM chart on P53 expression was shown (right). (**e**) Knockdown of MYCN enhanced the etoposide-induced P21 activation in HEL cells (*P*=0.036). (**f**) Overexpression of MYCN suppressed etoposide-mediated P21 activation in HEL and K562 cells (all *P*<0.001)

Previous studies revealed that MYC inactivation is associated with increased cellular senescence.^17, 18^ Thus, we hypothesized that MYCN suppresses senescence in erythroleukemia cells, thereby promoting their proliferation and tumorigenesis. Next, we measured the senescence characteristics, cell cycle progression and senescence-associated markers in MYCN-knockdown HEL cells. Suppression of MYCN resulted in increased senescence-associated acidic β-gal staining (Figure 3a). Cell cycle analysis showed an increased percentage of cells with G0/G1 phase and a decreased percentage of cells with S phase in the MYCN-knockdown cells (Figure 3b). Elevated activity of SA-β-gal and cell cycle arrested in G0/G1 phase are considered as the characteristics of cell senescence. To confirm further that MYCN depletion was resulting in cellular senescence, we examined the expression of additional molecular markers. P53 and P21 are widely thought as key regulators of cell senescence.^19^ We analyzed the expression of P21 and P53 using FCM. The results showed that depletion of MYCN elevated the P21 expression in HEL cells (Figure 3c and Supplementary Figure S4a). Interestingly, MYCN depletion did not induce an increase in P53 expression (Figure 3d and Supplementary Figure S4b). Previous study showed that treatment of etoposide could induce the P21 activation.^20^ In this study, FCM analysis showed that etoposide treatment resulted in an increase in P21 expression, and MYCN depletion enhanced the etoposide-induced P21 activation in HEL cells (Figure 3e and Supplementary Figure S4c). Further comparison analysis showed that overexpression of MYCN inhibited etoposide-mediated P21 activation in HEL and K562 cells (Figure 3f). These results indicate that MYCN counteracts P21-mediated cell senescence in a p53-independent transcription regulation.

### Identification of EZH2 as a target of MYCN

**Figure 4:**
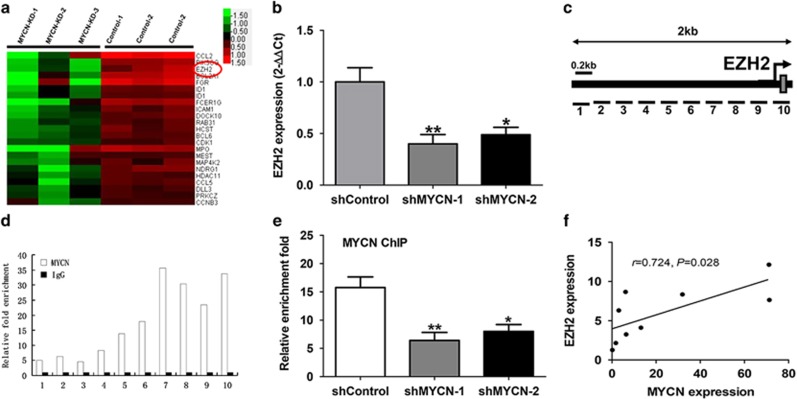
Identification of EZH2 as a target of MYCN. (**a**) Differential genes were screened through GEM analysis between the HEL cells with knockdown of MYCN and the control cells; EZH2 (signed by red circle) may be as a key gene targeted by MYCN. (**b**) Knockdown of MYCN decreased the EZH2 mRNA expression (*P*=0.004). (**c**) Ten pairs of primers, located sequentially along the proximal promoter and exon 1 (−1500bp to +200bp) of EZH2 were used to quantify the ChIP-enriched DNA. (**d**) ChIP-qPCR results showed that multiple MYCN-binding peaks were observed at a region near the transcriptional start site of EZH2. (**e**) Knockdown of MYCN resulted in a reduced enrichment of MYCN DNA binding to the EZH2 promoter (*P*=0.003). (**f**) The expression of MYCN had positive correlation with the expression of EZH2 in the patients with acute erythroleukemia (*P*=0.028)

In order to better understand the mechanism that MYCN promotes malignant phenotypes in leukemia cells, we tried to identify some key genes regulated by MYCN through gene expression microarray (GEM) analysis. The HEL cells with knockdown of MYCN and control cells were included in the GEM analysis to screen differential genes. The GEM results showed that 63 genes were up-regulated and 421 genes were downregulated in the cells with knockdown of MYCN compared with the control cells. The representative genes are listed in Figure 4a. Recent studies showed that MYC has been found to transcriptionally amplify epigenetic modifiers such as ezh2.^21, 22^ Considering the similar structure and function of MYC with MYCN as well as the driving effect of ezh2 on tumorigenesis, we focused on the effect of MYCN on EZH2 expression. The qPCR analysis showed that knockdown of MYCN significantly decreased the expression of EZH2 mRNA (Figure 4b). To investigate the regulatory mechanism of MYCN on EZH2, we evaluated whether MYCN can activate the activity of the EZH2 promoter in HEL cells. Ten pairs of primers, located sequentially along the proximal promoter and exon 1 (−1500 bp to +200 bp) of EZH2 were used to quantify the chromatin immunoprecipitation (ChiP)-enriched DNA (Figure 4c). The ChIP-qPCR results showed that MYCN-binding peaks were observed at a region nearby the transcriptional start site of EZH2. (Figure 4d). To further validate these data, we performed ChIP-qPCR in HEL cells transfected with knockdown of MYCN. The results showed that knockdown of MYCN led to significantly reduced enrichment of DNA binding in the EZH2 promoter (Figure 4e). Further qPCR analysis showed that the expression of MYCN had positive correlation with the expression of EZH2 in the patients with acute erythroleukemia (Figure 4f). All of these data indicate that MYCN directly activates EZH2 transcription by binding to its promoter.

### Overexpression of ezh2 reinforces malignant phenotypes in the HEL cells

**Figure 5:**
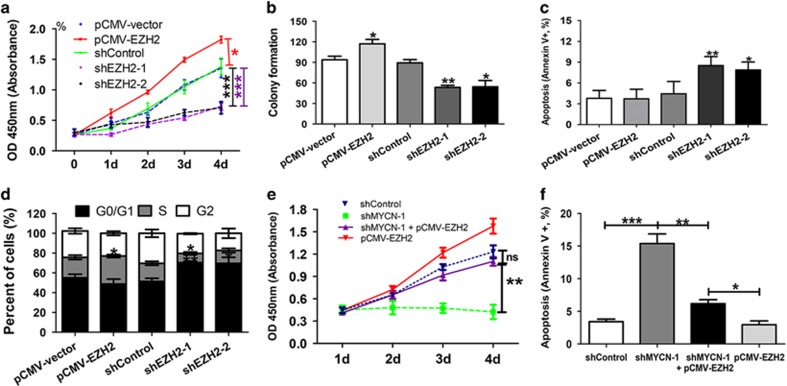
Overexpression of ezh2 reinforces malignant phenotypes in the HEL cells. (**a**) Overexpression of EZH2 resulted in a significant proliferation advantage (*P*=0.041), whereas knockdown of EZH2 reduced the cell growth compared with the control cells (*P*<0.001). (**b**) and (**c**) Overexpression of EZH2 led to an increase in colony formation, whereas knockdown of EZH2 decreased the colony formation and cell apoptosis compared with the control cells (all *P*<0.05). (**d**) Overexpression of EZH2 induced an incremental increase in the percentage of cells in S phase (*P*=0.045), whereas knockdown of EZH2 induced cell cycle arrested in G0/G1 phase (*P*=0.023). (**e**) and (**f**) The HEL cells with MYCN knockdown and EZH2 overexpression showed increased growth capacity and reduced cell apoptosis compared with the cells with MYCN knockdown, but did no difference in cell growth and apoptosis compared with the control cells

To define the role of EZH2 in leukemia cells, we constructed HEL with stable overexpression and knockdown of EZH2 using the lentivirus-mediated transfection system. The overexpression of EZH2 resulted in a significant increase in cell growth and colony formation (Figures 5a and b), whereas downregulation of EZH2 induced growth inhibition and increased cell apoptosis (Figure 5c). Overexpression of EZH2 induced an incremental increase in the percentage of cells with S phase, whereas knockdown of EZH2 induced cell cycle arrested in G0/G1 phase and reduced percentage of cells with S phase (Figure 5d). These data indicated that knockdown of EZH2 could produce similar biological effect to knockdown of MYCN. To investigate whether EZH2 overexpression antagonizes the biological effect of MYCN depletion, we co-transfected the shMYCN and pCMV-EZH2 lentivirus into HEL cells. Functional assays showed that overexpression of EZH2 could counteract the growth inhibition and cell apoptosis induced by knockdown of MYCN (Figures 5e and f,Supplementary Figure S5A and S5B). In addition, co-transfection led to reduced MYCN expression and rescued EZH2 expression (Supplementary Figure S5c and S5d).

### Depletion of MYCN induces p21 activation through removal of EZH2-mediated H3K27me3 on the p21 promoter

**Figure 6:**
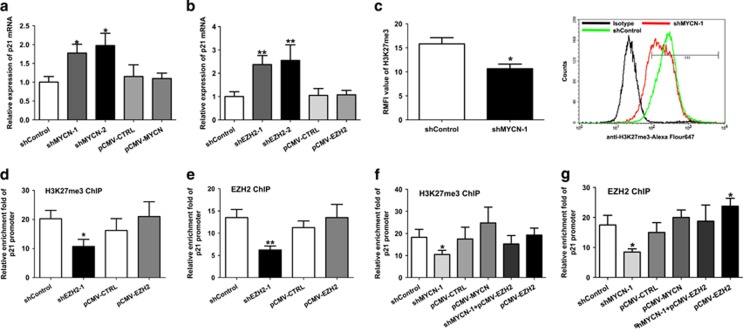
Depletion of MYCN induces p21 activation through removal of EZH2-mediated H3K27me3 on the p21 promoter. (**a**) Knockdown of MYCN in HEL cells caused apparent increase in p21 mRNA expression. (**b**) Knockdown of EZH2 also led to an increase in p21 mRNA expression. (**c**) Knockdown of MYCN resulted in apparent reduction of H3K27me3 level (*P*=0.013) (left). Representative FCM chart was shown (right). (**d**) Knockdown of EZH2 significantly reduced the enrichment level of H3K27me3 in the p21 promoter region (*P*=0.018). (**e**) EZH2 knockdown also reduced the enrichment level of EZH2 in the p21 promoter region (*P*=0.007). (**f**) Knockdown of MYCN significantly reduced the enrichment level of H3K27me3 in the p21 promoter region (*P*=0.026). Co-transfection of shMYCN with EZH2 overexpression moderately increased the enrichment level of H3K27me3 in the p21 promoter region compared with isolated shMYCN transfection. (**g**) Similarly, MYCN knockdown also decreased the enrichment level of EZH2 in the p21 promoter region (*P*=0.012), whereas co-transfection increased the enrichment level of EZH2 in the p21 promoter region compared with isolated shMYCN transfection

We next analyzed the mechanistic regulation involved in p21 expression by MYCN. Depletion of MYCN in HEL cells caused apparent increase in p21 mRNA expression (Figure 6a). Similarly, knockdown of EZH2 also led to an increase in p21 mRNA expression (Figure 6b). Neither MYCN overexpression nor EZH2 overexpression did affect the expression of p21 mRNA. It is well known that EZH2 regulates gene expression through H3K27 methylation modification. Thus, we hypothesized that depletion of MYCN may induce P21 activation through removal of EZH2-mediated H3K27 modification. FCM analysis showed that depletion of MYCN led to reduction of H3K27me3 (Figure 6c and Supplementary Figure S6a). To confirm the direct regulation between p21 and MYCN/EZH2 axis, we performed ChIP experiments using H3K27me3 antibody in the p21 promoter region. The ChIP results showed that depletion of EZH2 significantly reduced the enrichment level of H3K27me3 in the p21 promoter region (Figure 6d and Supplementary Figure S6b). EZH2 knockdown also reduced the enrichment level of EZH2 in the p21 promoter region (Figure 6e and Supplementary Figure S6c). Similarly, MYCN knockdown decreased the enrichment level of H3K27me3 in the p21 promoter region (Figure 6f and Supplementary Figure S6d). We also performed ChIP assay in the HEL cells co-transfected with shMYCN and pCMV-EZH2 lentivirus. However, co-transfection moderately increased the enrichment level of H3K27me3 in the p21 promoter region compared with sole shMYCN transfection. MYCN knockdown also decreased the enrichment level of EZH2 in the p21 promoter region, whereas co-transfection increased the enrichment level of EZH2 in the p21 promoter region (Figure 6g and Supplementary Figure S6e). Together, these results indicated that depletion of MYCN induces P21 activation through removal of EZH2-mediated H3K27me3 in the p21 promoter.

### MLN8237 induces the apoptosis of erythroleukemia cells while minimally affecting the normal hematopoietic cells

**Figure 7:**
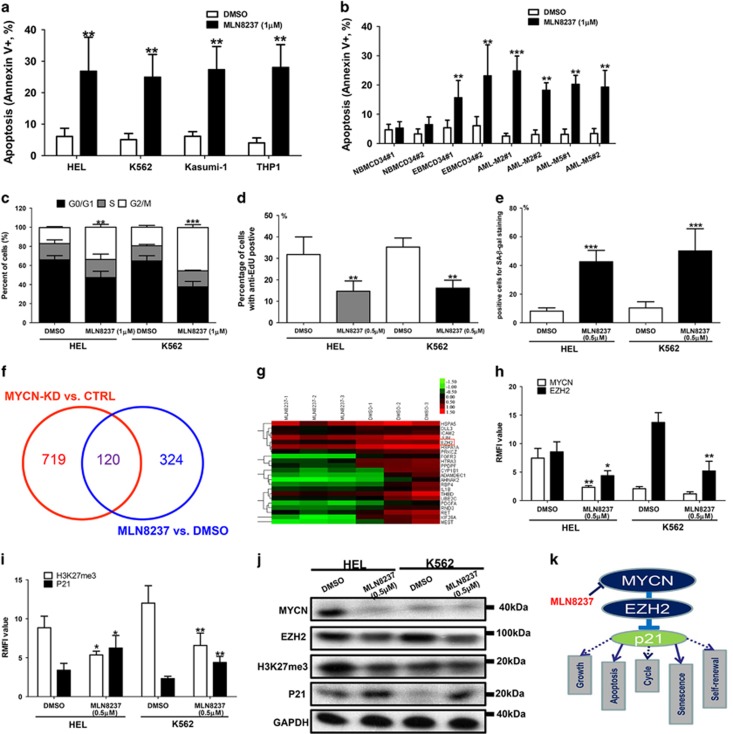
MLN8237 induces the apoptosis of erythroleukemia cells while minimally affecting the normal hematopoietic cells. (**a**) MLN8237 (1*μ*M) induced increased apoptosis in HEL, K562, Kasumi-1 and THP1 cells (all *P*<0.01). (**b**) MLN8237 (1*μ*M) induced obviously cell apoptosis in erythroleukemia CD34+ cells and other primary leukemia cells (all *P*<0.01). However, MLN8237 only induced mild cell apoptosis in normal CD34+ hematopoietic. (**c**) MLN8237 (1 *μ*M) induced significant G2/M phase block and decreased the percentage of cells in S phase in HEL (*P*=0.007) and K562 (*P*<0.001). (**d**) MLN8237 (0.5 *μ*M) obviously inhibited the cell growth of HEL (*P*<0.001) and K562 cells (*P*<0.001). (**e**) MLN8237 (0.5 *μ*M) treatment resulted in apparently increased percentage of senescence cells in HEL (*P*<0.001) and K562 (*P*<0.001) cells. (**f**) GEM analysis was performed in the HEL cells treated with MLN8237 or DMSO to identify some differential genes which are also regulated by MYCN. A total of 120 genes including EZH2 were differentially shared. (**g**) The representative cross genes are listed. (**h**) FCM analysis showed that MLN8237 (0.5 *μ*M) repressed the expression of MYCN and EZH2 (all *P*<0.05). (**i**) MLN8237 (0.5 *μ*M) treatment diminished the H3K27me3 level while activating the expression of P21 (all *P*<0.05). (**j**) WB analysis showed that MLN8237 (0.5 *μ*M) repressed the expression of MYCN, EZH2 and H3K27me3, whereas enhanced the expression of P21. (**k**) MYCN/EZH2 axis is critical for cell growth, anti-apoptosis, cycle progression, anti-senescence and self-renewal of leukemia cells through repression of p21 expression

It is well known that Aurora-A inhibitor MLN8237 can disrupt the Aurora-A/MYCN complex, promote degradation of MYCN and subsequently inhibit MYCN-dependent transcription.^23, 24^ In this study, MLN8237 induced increased apoptosis in HEL, K562, Kasumi-1, THP1, primary erythroleukemia CD34+ cells and primary AML-M2/M5 cells (Figures 7a and b). Cell apoptosis was induced by MLN8237 with a dose-dependent manner (Supplementary Figure S7a). However, MLN8237 only induced mild cell apoptosis in normal CD34+ hematopoietic cells (Figure 7b). MLN8237 also induced significant G2/M phase block in HEL, K562, Kasumi-1 and THP1 (Figure 7c and Supplementary Figure S7b). EdU uptake assay indicated that MLN8237 obviously inhibited the cell growth of those cell lines (Figure 7d and Supplementary Figure S7c). Primary erythroleukemia CD34+ cells and normal CD34+ hematopoietic cells were not involved in cell cycle and EdU uptake assay owing to almost all of these cells transitioning to the G0/G1 phase (quiescent phase). Besides, MLN8237 treatment resulted in apparently increased percentage of senescence cells in HEL and K562 cells (Figure 7e).

To explore the common mechanism shared by MYCN knockdown and MLN8237, we performed a GEM analysis in the HEL cells treated with MLN8237 or DMSO and tried to identify some differential genes which are also regulated by MYCN. Cross analysis showed that 120 genes including EZH2 were differentially shared (Figure 7f). The representative cross genes are listed in Figure 7g. FCM analysis showed that MLN8237 repressed the expression of MYCN, EZH2 and H3K27me3, whereas activated the expression of P21 (Figures 7h and i and Supplementary Figure S7d). Further validated WB analysis indicated similar results (Figure 7j). However, MLN8237 showed no influence on the expression of MYC and MYCL in leukemia cell lines (Supplementary Figure S7e and Figure S7f). Together, these data suggests that MLN8237 exerts its anti-leukemia effect through depletion of MYCN. In summary, MYCN/EZH2 axis is critical for cell growth, anti-apoptosis, cycle progression, anti-senescence and self-renewal of leukemia cells through repression of p21 expression (Figure 7k). MLN8237 can target this axis, and may be considered as a promising therapeutic agent.

## Discussion

MYC activation is frequent because of chromosomal translocations and transcriptional amplification in hematopoietic malignancies such as leukemia and lymphoma.^25, 26^ However, the role of MYCN has been poorly understood due to tissue restriction.^27^ Overexpression of MYCN was observed in adult leukemia patients from some studies with a small sample size.^14, 15^ Kawagoe *et al.*^15^ reported that overexpression of MYCN not MYC rapidly causes AML in mouse model, which emphases the driving effect of MYCN on leukomogenesis. Based on these findings, the functional mechanism needs to be further elucidated.

Previous studies have demonstrated the crucial role of MYC in erythroid differentiation block and erythroleukemia development.^5, 28^ In this study, we determined the expression of MYCN in erythroleukemia patients, and performed a series of functional experiments to investigate the effect of MYCN on biological characteristics of erythroleukemia cells. Higher MYCN expression was observed in erythroleukemia patients compared with normal controls, suggesting the potential role of MYCN in the maintenance of malignant characteristics of erythroleukemia. *In vitro* experiments, we observed that depletion of MYCN reduced cell growth and induced cell senescence. Further studies revealed that depletion of MYCN activated P21 expression in a P53-independent manner. Previous study indicated that knockdown of MYCN induced G0/G1 phase block together with increased expression of P21 in MYCN-overexpressed neuroblastoma cell lines.^29^ In general, p21 activation is mainly attributed to TP53 activation owing to its binding to the p21 promoter.^30^ However, in this study, homozygous p53 M133K mutation identified in HEL cells is located in p53 DNA-binding region, and severely impairs the transcriptional regulation of p53 on p21, which indirectly explained the reason for asynchronous expression between TP53 and P21. Hence, P21 activation may be possibly attributed to some P53-independent manners in MYCN knockdown cell with co-existing p53 mutation.

To establish the connection between MYCN and p21, we performed GEM in HEL cell line following MYCN knockdown. EZH2 was identified as a target of MYCN. Further ChIP results revealed that MYCN activates EZH2 transcription by binding to its promoters. MYC has been shown to induce EZH2 expression in embryonic stem cells and solid cancers,^21, 22, 31^ which is coincident with our results. Both MYCN and MYC collaborates EZH2 to maintain the PcG-mediated gene silencing.^32, 33^ Moreover, the role of EZH2 in leukemia cells was also investigated. Overexpression of EZH2 enhanced the malignant characteristics of leukemia cells, whereas downregulation of EZH2 diminished them. EZH2 overexpression can resist on the biological effect induced by MYCN knockdown. This antagonistic effect is incomplete because MYCN as a transcription factor does not only regulate the EZH2 transcription, but also regulates other cancer-related genes. These data indicated that MYCN contributes to the malignant characteristics of leukemia cells by stimulating EZH2 expression. The oncogenetic activity of EZH2 has been described in many solid cancers.^34, 35^ Overexpression of EZH2 is frequently detected in cancer tissues including lymphoma and leukemia and correlated with poor prognosis.^36, 37^ Several studies revealed that repression of EZH2 expression results in cellular senescence by inducing p21 and/or p16 activation.^38, 39, 40^ Mechanically, although p53-dependent transcription is the most common manner for p21 activation, p21 is also a common epigenetic target in cancer, with HDAC or H3K27-mediated repression of p21/CDKN1A occurring in cancer cells in a p53-independent manner.^39, 40^ Consistently, our study has also shown that knockdown of EZH2 or MYCN leads to epigenetic activation of p21 through removal of H3K27me3 in the p21 promoter. In HEL cells with p53 mutation, p21 is transcriptionally activated by depletion of EZH2 or MYCN independently of p53. However, overexpression of MYCN or EZH2 did not apparently inhibit the P21 expression, suggesting that MYCN/EZH2 axis is essential for inhibition of p21, whereas not for enhanced activation of p21. It is well known that p21 activation suppresses cell proliferation, induces cell apoptosis and cell cycle arrested into G0/G1 phase, and further leads to cellular senescence. Hence, we speculated that MYCN/EZH2 axis contributes to the malignant characteristics of erythroleukemia through H3K27me3-mediated epigenetic repression of p21.

Until now, no specific molecule targeting MYCN has been identified. However, the findings based on the collaboration between MYCN and other signaling proteins indirectly supply intervention strategies. MYCN binds with Aurora-A kinase to form a complex, which prevents Fbxw7-mediated proteasomal degradation of MYCN.^24, 41^ MLN8237 is an orally available selective inhibitor of Aurora-A kinase, and can disrupt the Aurora-A/MYCN complex and promote degradation of MYCN.^23, 24^ Disruption of the Aurora-A/MYCN complex inhibits MYCN-dependent transcription, which leads to tumor regression and prolonged survival in MYCN-driven cancer model.^24^ Aurora-A kinase is significantly overexpressed in the blasts in the AML patients compared with normal CD34+ cells.^42^ Overexpression of Aurora-A is associated with unfavorable cytogenetic abnormalities and high white blood cell number. Hence, we used MLN8237 to investigate its anti-leukemic activity in erythroleukemia cells. The results showed that MLN8237 treatment resulted in cell senescence and apoptosis in erythroleukemia cells. Further functional experiments revealed that MLN8237 treatment inhibits MYCN and EZH2 expression, and activates P21 expression, consistent with the biological effects caused by MYCN or EZH2 knockdown. Previous study revealed that MLN8237 induces cell apoptosis and senescence and inhibits cell proliferation through the upregulation of p21 and p27 expression in multiple myeloma.^43^ MLN8237 also impaired mitosis, induced senescence and markedly blocked proliferation in metastatic melanoma tumors.^44^ These results suggested a potential treatment possibility of MLN8237 for erythroleukemia. In fact, MLN8237 is currently in clinical testing in patients with advanced solid tumors, lymphoma and acute myelogenous leukemia.^45, 46, 47^ Preliminary results showed that 17% of AML patients (6/35) acquired treatment response.^47^ In general, our findings offer strategies to develop future therapies for MYCN-overexpressed erythroleukemia and other MYCN- dependent leukemia.

In summary, MYCN contributes to the malignant characteristics of erythroleukemia through EZH2-mediated repression of p21. MYCN may serve as a therapy target for the patients with acute erythroleukemia.

## Materials and methods

### Patients, cells and antibodies

A total of nine patients with acute erythroleukemia (AML-M6), eight patients with acute myeloblastic leukemia with maturation (AML-M2), six patients with acute monocytic leukemia (AML-M5), six patients with acute promyelocytic leukemia (AML-M3) and 29 cases with normal control were involved in this study. AML was diagnosed according to the FAB criteria.^48^ Erythroleukemia cell lines HEL and K562, M2 cell line Kasumi-1 and M5 cell line THP1 were obtained from ATCC, and maintained in complete medium (RPMI 1640 supplemented with 10% heat-inactivated fetal bovine serum) at 37 °C under 5% CO_2_. The research was approved by the ethics committee of the Shanghai Jiao tong University affiliated Sixth People’s Hospital. MYCN, EZH2, P21 and H3K27me3 antibodies for FCM were purchased from Abcam (Cambridge, MA, USA). Anti-P53-PE was purchased from BD Pharmingen (Shanghai, China).

### RNA preparation and qRT-PCR

The total RNA was extracted by using the RNeasy system (Qiagen, Valencia, CA, USA) following the manufacturer’s instructions, and the RNA was reversed transcribed into cDNA. The PCR reactions for the MYCN, MYC, MYCL, EZH2, HBG1, KLF1, p21 and GAPDH genes were performed using the ABI PRISM 7500 System (Applied Biosystems, Foster city, CA, USA) and SYBR Green Master Mix (Transgene, Beijing, China). The relative expression was calculated using 2Δ-ΔCT.

### Lentivirus-mediated cell transfection

MYCN-, EZH2- and control-shRNA were cloned into the pU6-MCS-Ubiquitin- EGFP-IRES-puromycin vector. Two shMYCNs and two shEZH2s were used in transfection experiments. A full-length CDS of the MYCN and EZH2 gene was cloned into the pCDH-CMV-copGFP vector. The lentivirus package was performed in HEK293T cells. Lentivirus (1–3 × 108 TU/ml) was transfected into the HEL and K562 cells. In brief, 5 × 105 cells/well in a six-well plate was incubated with the virus and polybrene (5 *μ*g/ml) in a 1 ml volume. The overexpression and the silencing efficiency were evaluated by using qRT-PCR and FCM.

### Intracellular flow cytometry assay

After treatment with a fixing solution and a permeabilizing solution, the cells were stained with a series of intracellular fluorescence antibodies at room temperature for 45 min. The expression of intracellular proteins was quantified based on the relative mean fluorescence intensity. A FACS Calibur equipped with the CellQuest software was used for logarithmic (Log) sampling.

### WST-1 cell proliferation assay

A total of 5 × 10^3^ cells (each well) were plated in 96-well plates in triplicate and incubated overnight. After culture, 10 *μ*l of WST-1 working solution (Keygen, Nanjing, China) was added to each well, and the cells were incubated for 2 h. The optical density (OD) at 450 nm was measured using a microplate reader. The growth curve was draw according to the OD value.

### EdU proliferation assay

For cell proliferation analysis by 5-ethynyl-2’- deoxyuridine (EdU), treated cells were incubated with EdU (20 *μ*M) for half hour in complete medium. After treatment with a fixing solution and a permeabilizing solution, the cells were stained with anti-EdU antibody. EdU staining was performed with a Click-iT Plus EdU Alexa Fluor 647 Flow Cytometry Assay Kit (Life Technologies, Shanghai, China), according to the manufacturer’s protocol. Stained cells were run on a FACS Calibur (BD Becton, Franklin Lakes, NJ, US). The data were analyzed using CellQuest software.

### Colony formation assay

Cells were plated in six-well plates with methylcellulose medium containing SCF, GM-CSF, IL-3 and erythropoietin (EPO; StemCell Technologies, Hangzhou, China) at 2 × 10^3^ cells/well in duplicate wells for each condition. After 14 days of incubation in a humidified incubator at 37°C, the colonies containing at least 30 cells were counted.

### Apoptosis detection

Annexin V-APC/7-AAD Apoptosis Kit (Keygen) was used to detect the apoptosis of cell lines quantitatively according to manufacturer’s instructions. Cells were incubated with anti-Annexin V-APC and 7-AAD for 10 min in the dark. FACS Calibur flowcytometer was used for detection of apoptotic cells. Annexin V-positive and PI-negative cells were considered to be apoptotic cells.

### Cell cycle analysis

Cells were fixed in 70% ethanol, washed with PBS once more, and then re-suspended in 1 ml of PI staining reagent. Samples were incubated in the dark for 30 min before cell cycle analysis. The cell cycle was measured with FACS Calibur. The percentages of cells in the G1, S and G2 phases were calculated with the Cellquest software.

### Erythroid differentiation

To induce erythroid differentiation, K562 and HEL cells were cultured with hemin 50 *μ*M or recombinant human EPO 3U/ml for 72 h. Erythroid differentiation was studied by analyzing the expression level of HBG1 and KLF1 using qRT-PCR. Expression of the erythroid markers CD71 and CD235a was evaluated by FCM.

### GEM

A GeneChip PrimeView Human Gene Expression Array (Affymetrix, Santa Clara, CA, USA) was used for the GEM study. The signal intensities were acquired with a GeneChip Scanner 3000 7G (Affymetrix) to generate cell intensity files. A robust multi-array average algorithm was used to normalize the data. The false discovery rate was <0.2, which minimizes the false identification of genes. The differential gene expression profiles were identified among the shRNA controls (*n*=3) and the MYCN knockdown (*n*=3) group.

### ChIP assay and ChIP-qPCR

ChIP assay kit (Upstate Biotechnology, Waltham, MA, USA) was used according to the manufacturer’s instructions. In brief, DNA was sheared to the lengths between 200 and 800 bp after fixation and lysis. The cross-linked protein was then immunoprecipitated using mouse anti-human MYCN, EZH2 or H3K27me3 monoclonal antibody (1:100) or non-specific IgG antibody (as the negative control of the antibody, Sigma, St. Louis, MO, USA). The DNA was extracted for PCR amplification. The DNA pool from ChIP, the input control and the IgG control were used for qPCR. PCR amplification was performed on an ABI 7500 real-time PCR machine (Applied Biosystems). The relative fold enrichment was calculated as %(ChIP/Input)/%(IgG control/Input).

### Statistical analysis

All statistical analyses were performed using the SPSS 21.0 System. Two independent samples were compared using the student's *t*-test. Multiple pairwise comparisons were made using a one-way analysis of variance. A *P*<0.05 was considered to be statistically significant.

### Western blotting

The primary antibodies included MYCN, EZH2, H3K27me3, P21 and GAPDH (Abcam Technology). Secondary human anti-rabbit antibody (Cell Signaling Technology, Danvers, MA, USA) conjugated to horseradish peroxidase was used. The detailed protocol is described in a previous study.^49^
