# Supplementary material for: MYCN contributes to the malignant characteristics of erythroleukemia through EZH2-mediated epigenetic repression of p21
Source: Cell Death Dis. 2017 Oct 12;8(10):e3126–. doi: 10.1038/cddis.2017.526 (PMC5682688; doi:10.1038/cddis.2017.526)
Supplement: Supplementary Figures [file cddis2017526x1.doc]

**
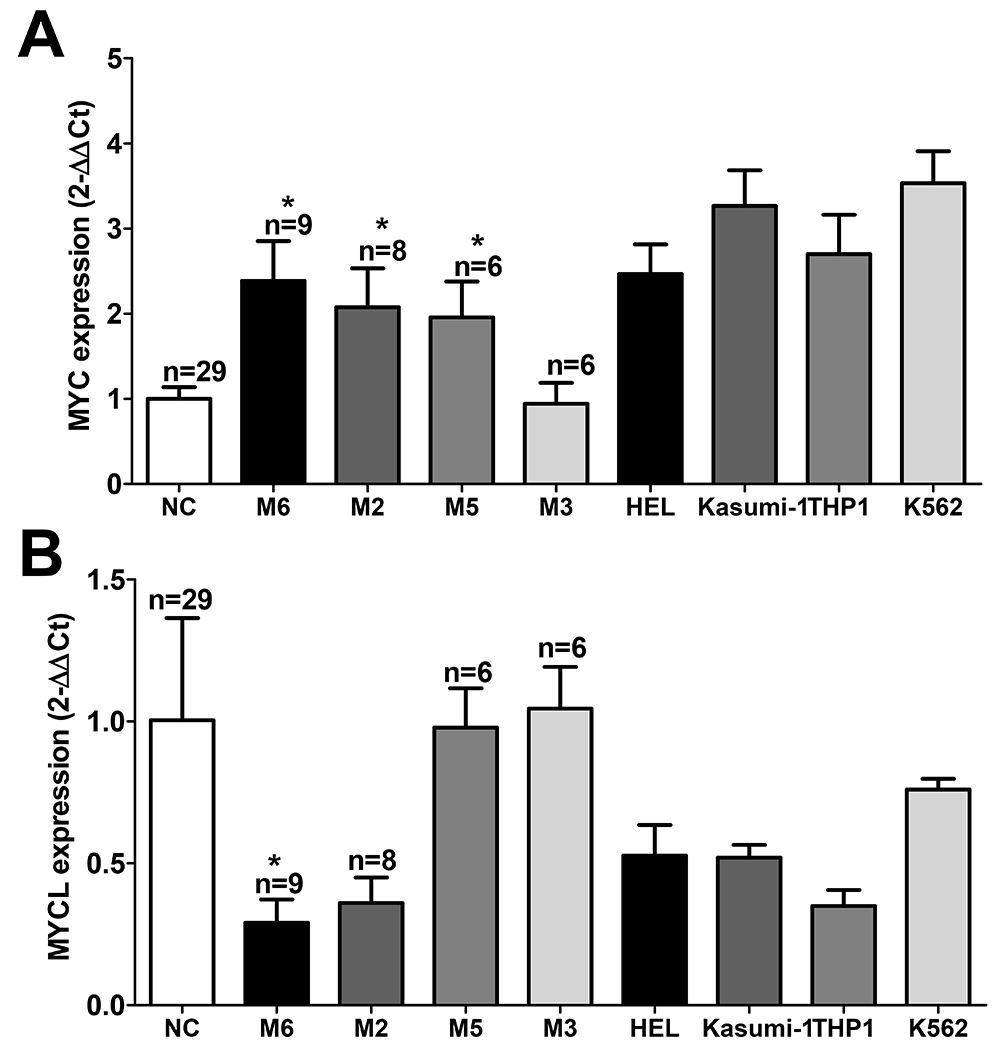
**

**Supplementary Figure. S1 Expression level of MYC and MYCL in leukemia patients and cell lines.**

(**a**) Higher MYC expression was found in the patients with M6, M2 and M5 compared with the normal controls (*P*=0.014; *P*=0.027; *P*=0.039). Leukemia cell lines including HEL, Kasumi-1, THP1 and K562 showed high expression of MYC. However, the patients with M3 don’t have high MYC expression. (**b**) The expression of MYCL was significantly reduced in the patients with M6 compared with the normal controls (*P*=0.011). *The corresponding statistical analysis relative to the control group is annotated with an asterisk. *, P<0.05; **, P<0.01; ***, P<0.001 (two-tailed, student T-test). Error bars throughout represent the SEM.*

**
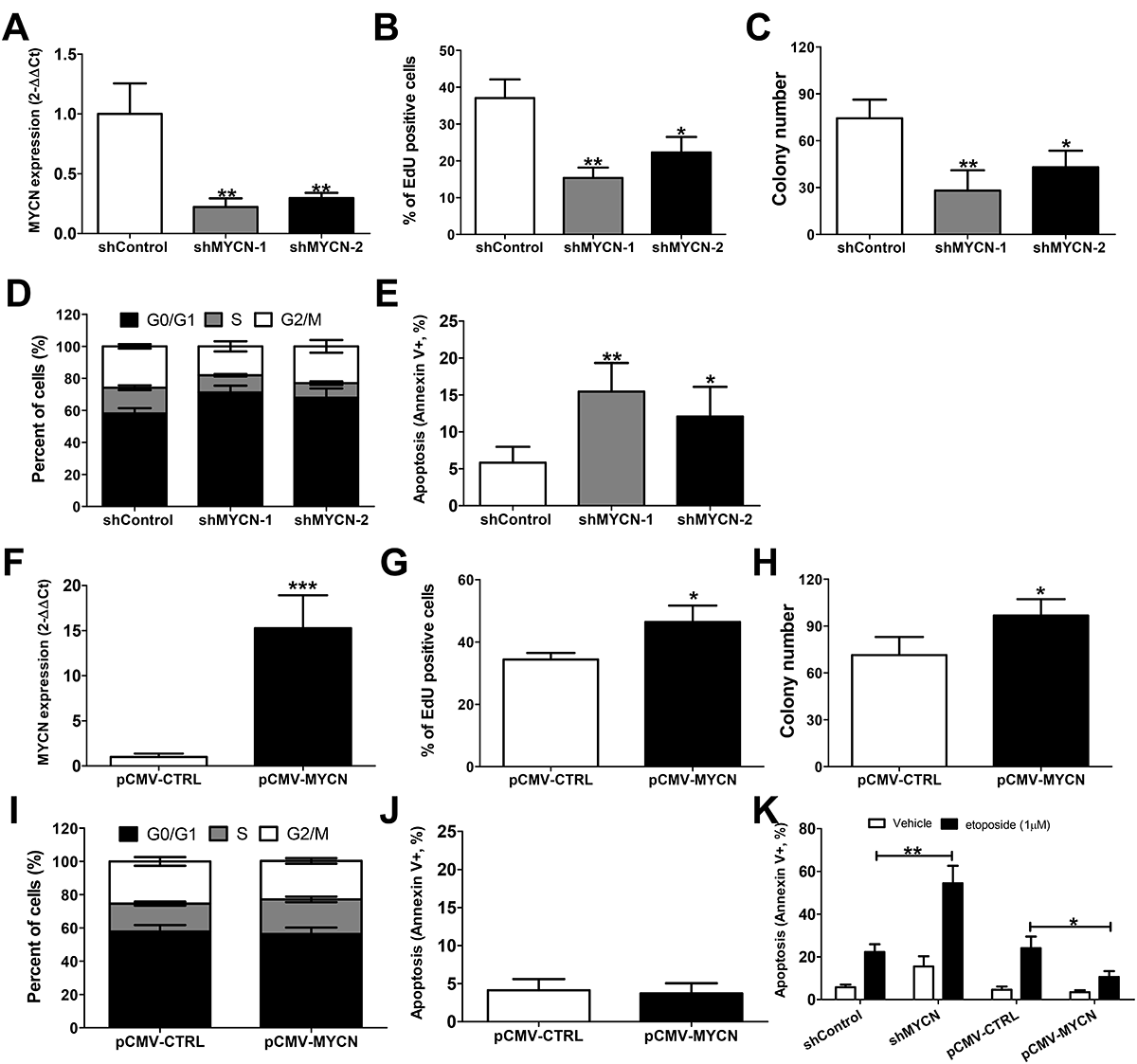
**

**Supplementary Figure. S2 MYCN overexpression promotes cell proliferation and enhances the resistance to etoposide-induced cell apoptosis in the Kasumi-1 cell line.**

(**a**) We constructed Kasumi-1 cells with stable knockdown of MYCN using lentivirus-mediated transfection. Suppression of MYCN inhibited cell proliferation (**b**), reduced the colony formation (**c**), induced cell cycle arrested into G0/G1 phase (**d**) and led to increased cell apoptosis (**e**). However, MYCN overexpression (**f**) promoted cell proliferation (**g**), increased the colony formation (**h**) (showed no effect on cell cycle and cell apoptosis, **i** and **j**) and reduced cell apoptosis sensitivity to etoposide (**k**).


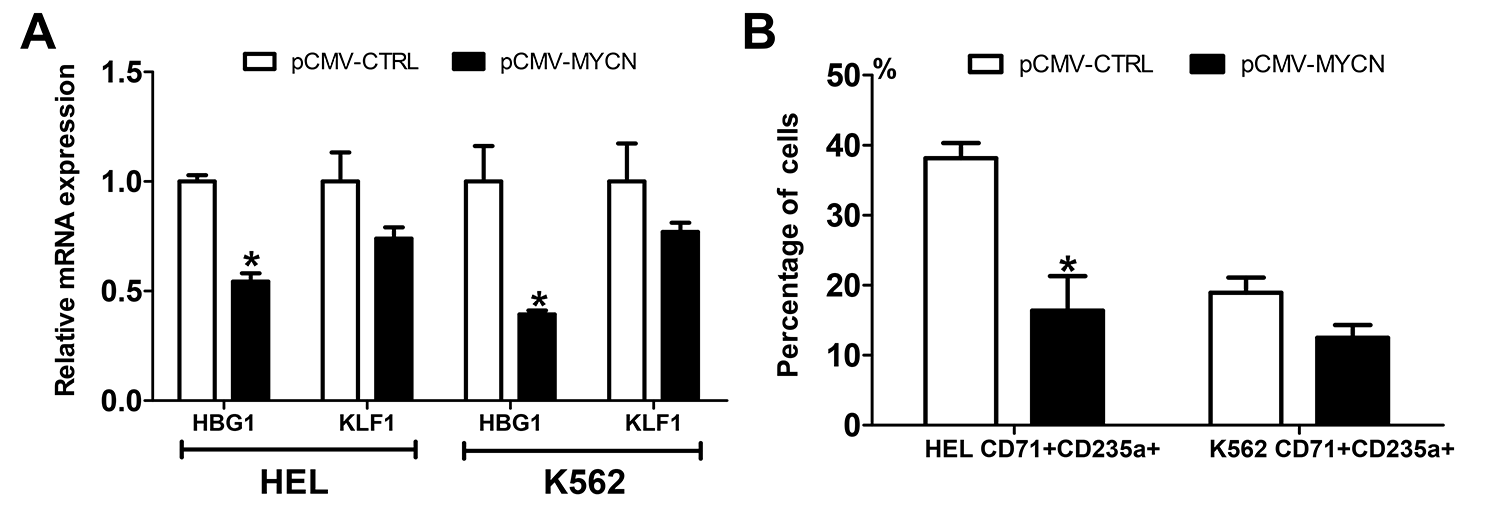


**Supplementary Figure. S3 MYCN overexpression inhibited EPO-induced erythroid differentiation.**

Co-culture of transfected cell lines with 3U/mL EPO for 72h was performed to induce erythroid differentiation. Erythroid differentiation markers HBG1 and KLF1 were determined using qRT-PCR. The expression of HBG1 and KLF1 was obviously reduced in HEL and K562 cells with MYCN overexpression compared with the control cells (**a**). In addition, MYCN overexpression led to a significant reduction in the percentage of EPO-induced CD71+CD235a+ erythroid populations in HEL and K562 cells (**b**).

**
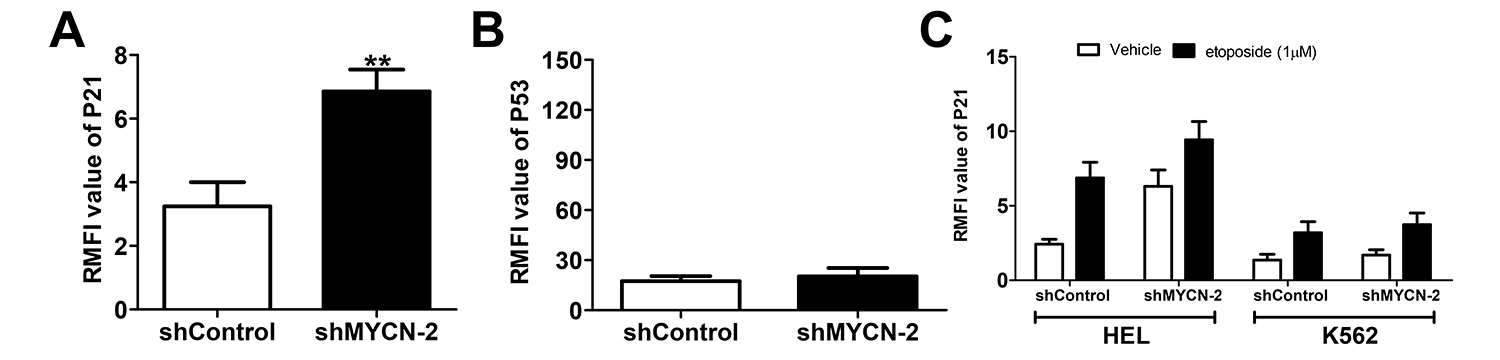
**

**Supplementary Figure. S4 Depletion of MYCN activates P21 expression in a p53-independent manner**

Functional experiments were performed using the second shMYCN. (**a**) Depletion of MYCN elevated the P21 expression in HEL cells. (**b**) MYCN depletion didn’t induce an increase in P53 expression. (**c**) MYCN depletion enhanced the etoposide-induced P21 activation in HEL cells.

**
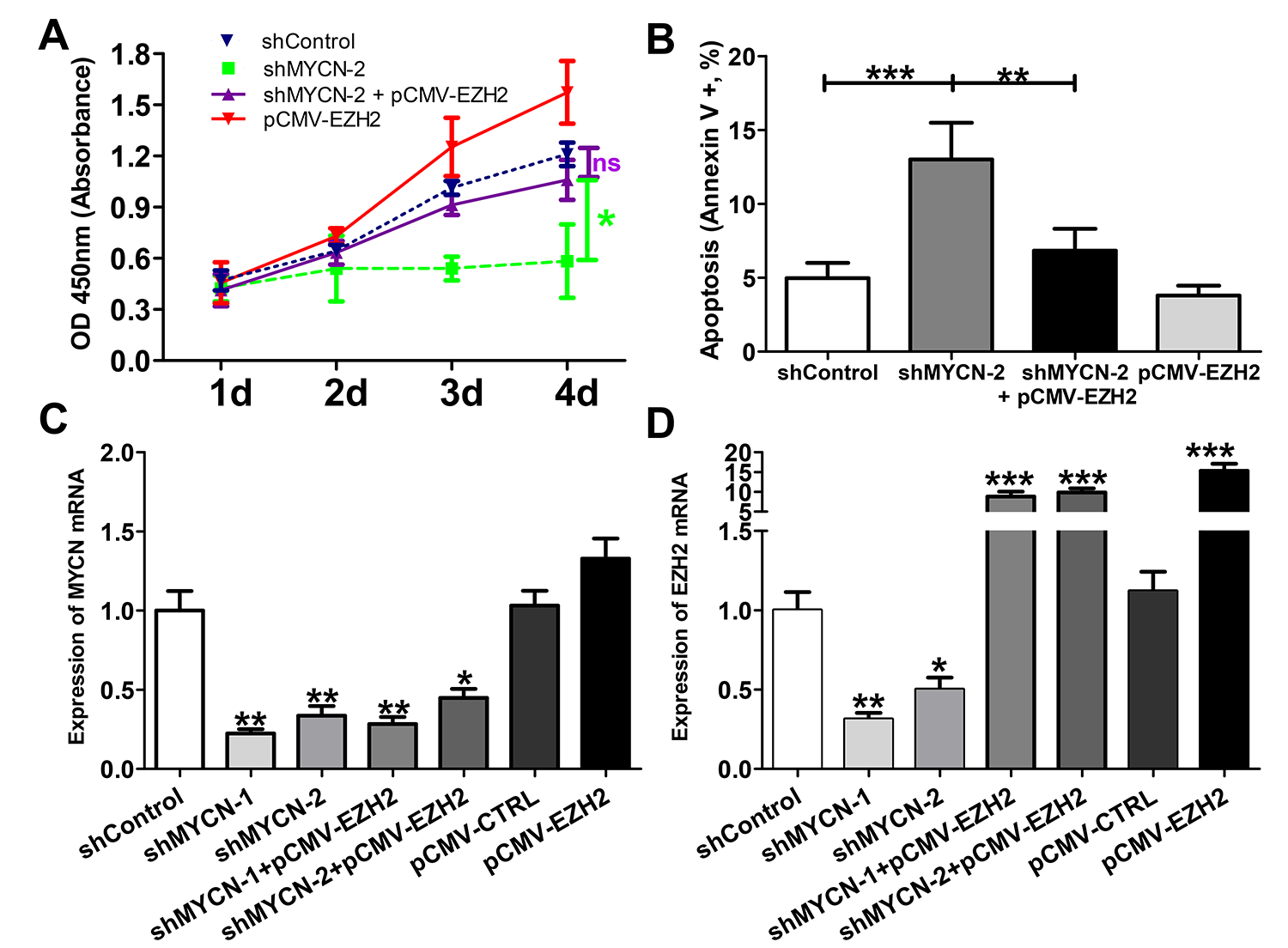
**

**Supplementary Figure. S5 Co-transfection of shMYCN and pCMV-EZH2 lentivirus in HEL cells.**

(**a**) and (**b**) Overexpression of EZH2 could counteract the growth inhibition and cell apoptosis induced by knockdown of MYCN. (**c**) Both isolated shMYCN transfection and co-transfection of shMYCN with pCMV-EZH2 led to reduced MYCN expression. (**d**) Isolated shMYCN transfection led to reduced EZH2 expression while co-transfection of shMYCN with pCMV-EZH2 resulted in a rescued EZH2 expression.

**
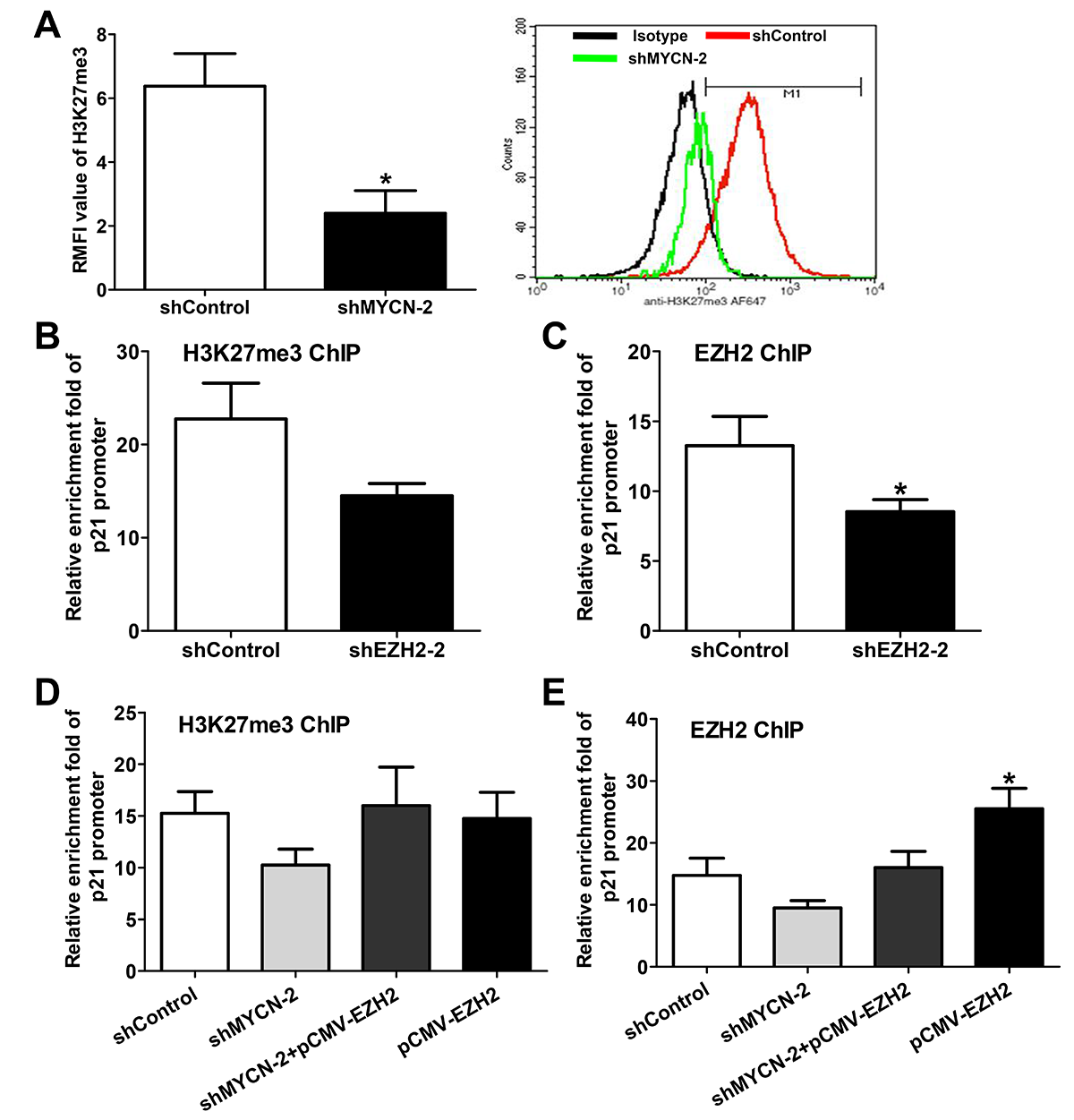
**

**Supplementary Figure. S6 Depletion of MYCN inhibited EZH2-mediated H3K27me3 on the p21 promoter**

Functional experiments were performed using the second shMYCN and shEZH2. (**a**) FCM analysis showed that depletion of MYCN led to reduction of H3K27me3 (left). Representative FCM chart was shown (right). (**b**) Depletion of EZH2 reduced the enrichment level of H3K27me3 in the p21 promoter region, although no significant difference was found. (**c**) EZH2 knockdown reduced obviously the enrichment level of H3K27me3 in the p21 promoter region. (**d**) MYCN knockdown decreased the enrichment level of H3K27me3 in the p21 promoter region. Co-transfected with shMYCN and pCMV-EZH2 lentivirus increased the enrichment level of H3K27me3 in the p21 promoter region compared with isolated shMYCN transfection. (**e**) MYCN knockdown also decreased the enrichment level of EZH2 in the p21 promoter region while co-transfection increased the enrichment level of EZH2 in the p21 promoter region compared with isolated shMYCN transfection.


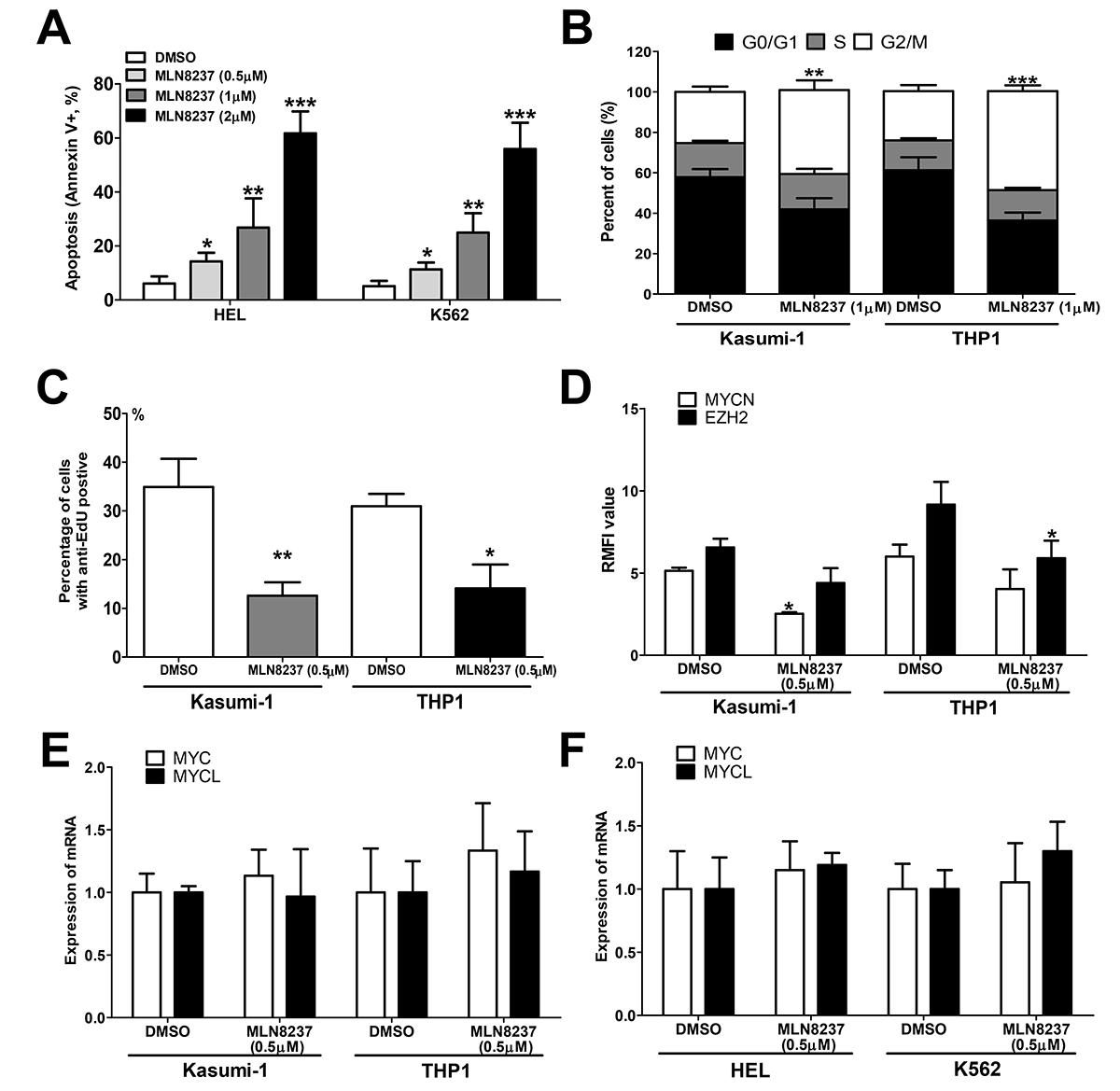


**Supplementary Figure. S7 The effect of MLN8237 on cell cycle, proliferation EZH2 and MYC family expression in leukemia cells.**

(**a**) MLN8237 induced cell apoptosis in HEL and K562 cells with a dose-dependent manner. (**b**) MLN8237 also induced significant G2/M phase block in Kasumi-1 and THP1. (**c**) MLN8237 obviously inhibited the cell proliferation in Kasumi-1 and THP1. (**d**) FCM analysis showed that MLN8237 repressed the expression of MYCN and EZH2 in Kasumi-1 and THP1 cells. (**e**) MLN8237 showed no influence on the expression of MYC and MYCL in Kasumi-1 and THP1 cells. (**f**) MLN8237 didn’t affect the expression of MYC and MYCL in HEL and K562 cells.
